# Genetics of morphological hip abnormalities and their implications for osteoarthritis: a scoping review

**DOI:** 10.1093/jhps/hnaf020

**Published:** 2025-04-18

**Authors:** Lainey G Bukowiec, Elizabeth S Kaji, John A Koch, Sami Saniei, Miguel M Girod-Hoffmann, Jason P Sinnwell, Cody C Wyles

**Affiliations:** Mayo Clinic Department of Orthopedic Surgery, 200 First St, Rochester, MN 55905, United States; Mayo Clinic Orthopedic Surgery Artificial Intelligence Lab, 200 First St, Rochester, MN 55905, United States; Mayo Clinic Orthopedic Surgery Artificial Intelligence Lab, 200 First St, Rochester, MN 55905, United States; Mayo Clinic Orthopedic Surgery Artificial Intelligence Lab, 200 First St, Rochester, MN 55905, United States; Mayo Clinic Orthopedic Surgery Artificial Intelligence Lab, 200 First St, Rochester, MN 55905, United States; Mayo Clinic Orthopedic Surgery Artificial Intelligence Lab, 200 First St, Rochester, MN 55905, United States; Mayo Clinic Department of Quantitative Health Sciences, 200 First St, Rochester, MN 55905, United States; Mayo Clinic Department of Orthopedic Surgery, 200 First St, Rochester, MN 55905, United States; Mayo Clinic Orthopedic Surgery Artificial Intelligence Lab, 200 First St, Rochester, MN 55905, United States

## Abstract

Morphological hip abnormalities (MHAs) significantly influence lifelong prognosis of the hip, contributing to early-onset osteoarthritis and impaired functionality. Developmental dysplasia of the hip (DDH) and femoroacetabular impingement (FAI) represent key pathologies, resulting from insufficient or excessive femoral head coverage, respectively. These abnormalities alter hip biomechanics, leading to structural damage, pain, and accelerated joint degeneration. Advances in genetic research have illuminated the interplay between genetics and mechanical loading in shaping hip morphology. Genes associated with osteoarthritis, DDH, and FAI include *COL1A1, MMP13*, and *IL-6*. Genes associated with FAI and osteoarthritis include *ADAMTS4*. Genes associated with DDH and osteoarthritis include *FRZB, CX3CR1, ASPN, DKK1, PDRG1, GDF5*, *UQCC1*, and *TGF-β1*. The mechanisms linking morphological derangements to symptomatic osteoarthritis remain incompletely understood. Multimodal approaches integrating imaging, biomechanics, and genetics may uncover distinct disease subtypes, enabling personalized interventions. Early detection of MHAs is critical in preventing early-onset osteoarthritis. Incorporating advanced imaging techniques, such as statistical shape modelling, can enhance the understanding of complex 3D hip morphologies and their progression to osteoarthritis. Future research should explore the genetic underpinnings of other morphologic hip conditions, including Slipped Capital Femoral Epiphysis and Legg–Calvé–Perthes disease, to refine preventive and therapeutic strategies. A comprehensive approach combining genetics, imaging, and clinical insights holds promise for mitigating the lifelong impact of MHAs.

## Introduction

**Figure 1.**
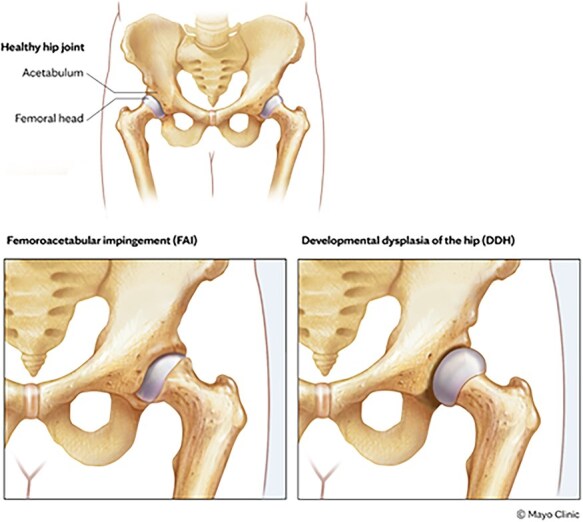
Normal and abnormal morphology of the hip joint. Used with permission of Mayo Foundation for Medical Education and Research, all rights reserved.

Morphological hip abnormalities (MHA) have critical implications for the lifelong prognosis of the hip joint. MHAs can manifest early in life as a painful hip with limited functionality and later in life as osteoarthritis. Morphological phenotypes exist on a spectrum of insufficient to excessive coverage of the femoral head, leading to local cumulative mechanical overload, structural damage, pain, and joint degeneration. The most influential recent insight in osteoarthritis aetiology has been the ‘integrated mechanical concept’ proposed by Ganz *et al*. [1] A morphologically normal hip strikes a compromise between congruence and constraint, enabling a lifetime of high function in most patients. In contrast, the hip joint can have too little congruence and constraint, as seen in developmental dysplasia of the hip (DDH), or too much constraint, resulting in femoroacetabular impingement (FAI). DDH and FAI can stem from the acetabulum, femur, or both. Complicating matters further, patients can also present with a ‘mixed’ morphology demonstrating features of DDH and FAI (Fig. 1).

Unlike degenerative diseases of other major joints, hip degeneration may be potentiated by structural pathologies of childhood and early adulthood [2, 3]. These pathologies share a common pathway of altering hip morphology, resulting in abnormal hip biomechanics for the remainder of a patient’s life. In the Rotterdam prospective study cohort consisting of >4000 patients without osteoarthritis at baseline, cam morphology and acetabular dysplasia led to a two-fold increase in the development of osteoarthritis at an average 9-year follow-up [4]. Clohisy *et al*. retrospectively examined 337 hips with idiopathic osteoarthritis that underwent total hip arthroplasty (THA) before age 51 and found prior radiographic features of DDH in 48.4%, FAI in 35% (22.5% with cam deformity, 2.1% with Pincer deformity, 10.4% with mixed pathology), Legg–Calvé–Perthes disease in 9.5%, and Slipped Capital Femoral Epiphysis (SCFE) in 6.2% [5]. Thomas *et al*. examined a prospective cohort of 1003 female patients and found that cam deformity and mild acetabular dysplasia were predictive of end-stage osteoarthritis requiring THA, independent of age, BMI, and joint space [6]. Additionally, more severe cam deformities have been associated with a heightened risk of rapid progression to end-stage hip osteoarthritis [6, 7].

### Aetiology of DDH

The aetiology of DDH is multifactorial, involving environmental and genetic factors. Risk factors include female sex, breech presentation, oligohydramnios, primiparity of the pregnancy, high birth weight, and swaddling [8]. DDH generally follows an autosomal dominant inheritance pattern with incomplete penetrance [9]. Monozygotic twins have a 41% increased risk of DDH compared to dizygotic twins [10]. Population studies indicate a 9- to 12-fold increase in DDH risk for first-degree relatives and siblings [11, 12]. Variability in the incidence of DDH also suggests a strong genetic component, with rates ranging from 0.06 to 76.1 per 1000 live births in African and Native American populations, respectively. Notably, there is considerable diversity in incidence rates within each racial group, influenced by geographic location [13].

### Aetiology of FAI

Like DDH, the development of FAI is influenced by both genetic and environmental factors. It has been shown that participation in high-intensity sports during adolescence is associated with a higher prevalence of cam deformity, suggesting that lifestyle factors influence the development of this condition [14]. There is also evidence to support a genetic component, as the prevalence of FAI varies between Asian and white populations [15, 16]. Furthermore, siblings of patients with FAI have a nearly three-fold increase in the prevalence of cam deformity and clinical symptoms (relative risk of 2.8 and 2.5, respectively) than control subjects [17]. Multiple studies have suggested a strong association of cam deformity with hip osteoarthritis [5–7, 18, 19].

### Role of genetics

Genetic biomarkers may serve as early indicators of MHAs [20]. While current notions emphasize the impact of mechanical loading on morphological phenotype, the specific genetic loci contributing to hip morphology variations and their association with osteoarthritis remain elusive. Recent advancements highlight the complex polygenic nature of hip morphogenesis [21–23]. Genetic markers associated with modest effect sizes for individual genes have been identified, yet the mechanistic pathway underlying why some hips with MHA progress to symptomatic osteoarthritis while others do not remains unclear [24, 25]. Genetic studies that integrate imaging and well-defined phenotyping of patients with MHAs can provide valuable insights into important pathways and potential personalized therapeutic targets for early intervention to mitigate the long-term impact of MHAs on hip joint health.

Genome-wide association studies (GWAS) have proven invaluable in uncovering genetic variations, particularly single-nucleotide polymorphisms (SNPs), that are associated with musculoskeletal disorders and anatomical variants [26]. However, the modest effects of individual SNPs identified by GWAS often obscure their broader morphological implications. To address these gaps, the current study extends beyond traditional GWAS by incorporating a comprehensive approach that includes not only GWAS but also whole exome sequencing (WES) and other modalities. This multi-faceted approach allows for a deeper exploration of the genetic and phenotypic relationships, providing a more nuanced understanding of the genetic factors contributing to musculoskeletal conditions and their progression.

## Methods

A comprehensive search of PubMed from inception to 23 May 2024, any language, was conducted. Only indexed publications were included. Controlled vocabulary supplemented with keywords was used to search for articles related to genetics and hip morphological conditions including DDH and FAI (see Appendix). Animal studies, case reports, and articles that covered rare, syndromic pathologies were excluded from the review (51, 1, and 36 articles excluded, respectively). Seven additional articles were excluded due to irrelevance as determined by agreement by three reviewers (L.G.B., E.S.K., and J.A.K.). There were 12 review articles, 1 meta-analysis, and 1 editorial that populated from the search. These articles were reviewed for overarching content to supplement findings with pertinent content. The initial query yielded 146 results (see Appendix). A total of 95 articles were excluded, leaving 51 publications for evaluation.

## Results

The search yielded articles focusing on the genetics of FAI, DDH, and osteoarthritis. Notably, several studies explored the genetic overlap between DDH and osteoarthritis, as well as between FAI and osteoarthritis, suggesting shared genetic pathways. Additionally, a few studies employed statistical shape modelling (SSM) to holistically examine hip morphology, categorizing various shapes and their implications for osteoarthritis and fracture risk [27–29]. While these comprehensive shape analyses were not the primary focus of our review, they inform some aspects of our discussion by providing a broader context of morphological influences on hip pathology. Important genes related to DDH, FAI, and their implications for osteoarthritis are discussed. Additional study findings including all mentioned genes are listed in Tables 1 and 2.

**Table 1.** Genes implicated in DDH from literature review.

Chromosome	Gene(s); location	Study	Inheritance details [62]	Study type	Method	Population
1	*COL11A1*; 1p21.1, rs3753841, rs145901197	Sun *et al*. [76]	Familial and sporadic	Cross-sectional	GWAS + replication and WES	GWAS conducted on Chinese population: 3922 healthy, 1256 DDH cases; WES conducted on Saudi-Arabian, Chinese populations
	*NOTCH2*; 1p12	Dembic *et al*. [77]	Familial	Cross-sectional	WES	Danish population: 29 families examined including 66 DDH cases
	*CDC7*; 1p22.1 *TGF-β2*; 1q41	Li *et al*. [64]	Sporadic	Cross-sectional	Biological assays including cell cycle, viability, apoptosis, immunofluorescence, RT-PCR,^a^ and western blotting	Chinese population: unilateral and bilateral 15 DDH cases and 12 healthy
	*HSPG2*; 1p36.12 *ATP2B4*; 1q32.1	Basit *et al*. [78]	Familial	Cohort	WES	Four individuals of a family with DDH
	*NOTCH2*; 1p12	Dembic *et al*. [77]	Familial	Cross-sectional	WES	Danish population: 29 families examined including 66 DDH cases
2	*FN1*; 2q35 *COL6A3*; 2q37.3	Zhao *et al*. [66]	Sporadic	Cross-sectional	Transcriptome analysis and single-cell RNA sequencing	Ligamentum teres tissue samples from six DDH cases undergoing open reduction surgery
	*DIS3L2*; 2q37.1	Dembic *et al*. [77]	Familial	Cross-sectional	WES	Danish population: 29 families examined including 66 DDH cases
	*FRZB*; 2q32.1	Yang *et al*. [79]	Sporadic	Cross-sectional	Bioinformatics	Not specified
	*COL3A1*; 2q32.2	Li *et al*. [64]	Sporadic	Cross-sectional	Biological assays including cell cycle, viability, apoptosis, immunofluorescence, RT-PCR, and western blotting	Chinese population: unilateral and bilateral 15 DDH cases and 12 healthy
	*HOXD9*; 2q31.1 and rs711819	Tian *et al*. [80]	Sporadic	Case–control	SNP genotyping with matrix-assisted laser desorption ionization–time-of-flight mass spectrometry	Han Chinese population: 209 DDH cases and 173 healthy
3	*EPHA6*; 3q11.2	Zhao *et al*. [66]	Sporadic	Cross-sectional	Transcriptome analysis and single-cell RNA sequencing	Ligamentum teres tissue samples from six DDH cases undergoing open reduction surgery
	*TM4SF19*; 3q29	Dembic *et al*. [77]	Familial	Cross-sectional	WES	Danish population: 29 families examined including 66 DDH cases
	*CX3CR1*; 3p22.2 (rs3732378 discussed in the Gumus paper)	Yang *et al*. [79]	Sporadic	Cross-sectional	Bioinformatics	Not specified
		Gumus *et al*. [34]	Sporadic	Cross-sectional	RT-PCR	Turkish population: 68 DDH cases and 100 healthy
4	*EVC2*; 4p16.2	Dembic *et al*. [77]	Familial	Cross-sectional	WES	Danish population: 29 families examined including 66 DDH cases
	*TENM3*; 4q34.3-q35.1	Yang *et al*. [79]	Sporadic	Cross-sectional	Bioinformatics	Not specified
	*CCNA2*; 4q27	Li *et al*. [64]	Sporadic	Cross-sectional	Biological assays including cell cycle, viability, apoptosis, immunofluorescence, RT-PCR, and western blotting	Chinese population: unilateral and bilateral 15 DDH cases and 12 healthy
	*UFSP2*; 4q35.1 and rs7663196	Watson *et al*. [81]	Familial	Cross-sectional	Sanger sequencing	South African family of European origin
5	*CCNB1*; 5q13.2	Li *et al*. [64]	Sporadic	Cross-sectional	Biological assays including cell cycle, viability, apoptosis, immunofluorescence, RT-PCR, and western blotting	Chinese population: unilateral and bilateral 15 DDH cases and 12 healthy
6	*WISP3*; 6q22	Yang *et al*. [79]	Sporadic	Cross-sectional	Bioinformatics	Not specified
7	*COL1A2*; 7q21.3	Zhao *et al*. [66]	Sporadic	Cross-sectional	Transcriptome analysis and single-cell RNA sequencing	Ligamentum teres tissue samples from six DDH cases undergoing open reduction surgery
	*IL-6*; 7p15.3	Yang *et al*. [79]	Sporadic	Cross-sectional	Bioinformatics	Not specified
	*TXNDC3*; 7p14.1 and rs10250905	Qiao *et al*. [82]	Sporadic	Case–control	RT-PCR	Han Chinese population: 984 DDH patients and 2043 healthy controls
8	*CTSB*; 8p23.1 *FDFT1*; 8p23.1	Mori *et al*. [83]	Sporadic	Cross-sectional	GWAS + replication study, GSEA^b^, transcriptome analysis, RT-PCR, and genetic association analysis	GWAS conducted on Japanese population: 238 cases and 2044 healthy; GWAS conducted on UK population: 3315 cases and 74038 controls
	*CCNE2*; 8q22.1	Li *et al*. [64]	Sporadic	Cross-sectional	Biological assays including cell cycle, viability, apoptosis, immunofluorescence, RT-PCR, and western blotting	Chinese population: unilateral and bilateral 15 DDH cases and 12 healthy
9	*PTPRD*; 9p24.1-p23 *COL5A1*; 9q34.4	Zhao *et al*. [66]	Sporadic	Cross-sectional	Transcriptome analysis and single-cell RNA sequencing	Ligamentum teres tissue samples from six DDH cases undergoing open reduction surgery
	*SHC3*; 9q22.1	Dembic *et al*. [77]	Familial	Cross-sectional	WES	Danish population: 29 families examined including 66 DDH cases
	*ASPN*; 9q22.31	Yang *et al*. [79]	Sporadic	Cross-sectional	Bioinformatics	Not specified
10	*DKK1*; 10q21.1	Yang *et al*. [79]	Sporadic	Cross-sectional	Bioinformatics	Not specified
	*CDK1*; 10q21.2	Li *et al*. [64]	Sporadic	Cross-sectional	Biological assays including cell cycle, viability, apoptosis, immunofluorescence, RT-PCR, and western blotting	Chinese population: unilateral and bilateral 15 DDH cases and 12 healthy
11	*OTOG*; 11p15.1	Dembic *et al*. [77]	Familial	Cross-sectional	WES	Danish population: 29 families examined including 66 DDH cases
	*MMP1, MMP3*, and *MMP13*; 11q22.2	Li *et al*. [64]	Sporadic	Cross-sectional	Biological assays including cell cycle, viability, apoptosis, immunofluorescence, RT-PCR, and western blotting	Chinese population: unilateral and bilateral 15 DDH cases and 12 healthy
	*MMP9*; 20q13.12					
	*WNT11*; 11q13.5					
	*HSPA8*; 11q24.1	Yan *et al*. [84]	Sporadic	Case–control	GWAS	A total of 960 DDH patients and 1069 controls
12	*METTL21B*; 12q13	Dembic *et al*. [77]	Familial	Cross-sectional	WES	Danish population: 29 families examined including 66 DDH cases
	*LRP1*; 12q13.3	Yan *et al*. [85]	Familial and sporadic	Cross-sectional	WES	Chinese population: 8 families with 17 DDH cases and 68 sporadic patients
	*COL2A1* and *VDR*; 12q13.11	Rubini *et al*. [86]	Familial	Case–control	GWAS	Eleven DDH families including 41 DDH cases and 73 healthy
		Granchi *et al*. [87]	Sporadic	Case–control	PCR	Caucasian population: 143 consecutive hip replacements and 50 healthy controls
		Wang *et al*. [88]	Sporadic	N/A	RT-PCR	Twelve donor surgical patients with or without DDH
13	*POSTN*; 13q13.3	Zhao *et al*. [66]	Sporadic	Cross-sectional	Transcriptome analysis and single-cell RNA sequencing	Ligamentum teres tissue samples from six DDH cases undergoing open reduction surgery
	*DACH1*; 13q21.33	Dembic *et al*. [77]	Familial	Cross-sectional	WES	Danish population: 29 families examined including 66 DDH cases
14	*GCH1*[88]; 14q22.2	Mori *et al*. [83]	Sporadic	Cross-sectional	GWAS + replication study, GSEA,transcriptome analysis, RT-PCR, and genetic association analysis	GWAS conducted on Japanese population: 238 cases and 2044 healthy; GWAS conducted on UK population: 3315 cases and 74 038 controls
15	*SMAD3*; 15q22.33	Li *et al*., 2021[64]	Sporadic	Cross-sectional	Biological assays including cell cycle, viability, apoptosis, immunofluorescence, RT-PCR, and western blotting	Chinese population: unilateral and bilateral 15 DDH cases and 12 healthy
	15q13.3	Basit *et al*. [36]	Familial	Cohort	Sanger sequencing of all DDH-associated genes, whole-genome SNP genotyping, and exome sequencing	Saudi family: four DDH cases, two parents, and two healthy
	*ACAN*; 15q26.1	Wang *et al*. [88]	Sporadic	N/A	RT-PCR	12 donor surgical patients with or without DDH
17	*COL1A1*; 17q21.33	Zhao *et al*. [66]	Sporadic	Cross-sectional	Transcriptome analysis and single-cell RNA sequencing	Ligamentum teres tissue samples from six DDH cases undergoing open reduction surgery
		Yang *et al*. [79]	Sporadic[66]	Cross-sectional	Bioinformatics	Not specified
		Li *et al*. [64]	Sporadic	Cross-sectional	Biological assays including cell cycle, viability, apoptosis, immunofluorescence, RT-PCR, and western blotting	Chinese population: unilateral and bilateral 15 DDH cases and 12 healthy
	*MYH10*; 17p13.1	Dembic *et al*. [77]	Familial	Cross-sectional	WES	Danish population: 29 families examined including 66 DDH cases
	*TBX4*; 17q23.2					
	*CDC6*; 17q21.2	Li *et al*. [64]	Sporadic	Cross-sectional	Biological assays including cell cycle, viability, apoptosis, immunofluorescence, RT-PCR, and western blotting	Chinese population: unilateral and bilateral 15 DDH cases and 12 healthy
	*HOXB9*; 17q21.32 and rs16949053	Feldman *et al*. [89]	Familial	Cross-sectional	GWAS	North American family, three generations
	*SOX9*; 17q24.3	Wang *et al*. [88]	Sporadic	N/A	RT-PCR	12 donor surgical patients with or without DDH
19	*TGF-β1*; 19q13.2	Yang *et al*. [79]	Sporadic	Cross-sectional	Bioinformatics	Not specified
		Li *et al*. [64]	Sporadic	Cross-sectional	Biological assays including cell cycle, viability, apoptosis, immunofluorescence, RT-PCR, western blotting	Chinese population: unilateral and bilateral 15 DDH cases and 12 healthy
		Basit *et al*. [36]	Familial	Cohort	Sanger sequencing of all DDH-associated genes, whole-genome SNP genotyping, and exome sequencing	Saudi family: four DDH cases, two parents, and two healthy
20	*GDF5*; 20q11.22 (rs143383 noted in Harsanyi papers)	Yang *et al*. [79]	Sporadic	Cross-sectional	Bioinformatics	Not specified
		Harsanyi *et al*. [90]	Sporadic	Cross-sectional	RT-PCR	Caucasian population: 59 DDH cases and 59 healthy
		Harsanyi *et al*. [91]	Sporadic	Cross-sectional	CGAA	Slovakian population: 45 DDH cases and 85 healthy
		Sadat-Ali *et al*. [45]	Sporadic and familial	Case–control	CGAA	100 DDH patients, 200 parents, 73 siblings, and 100 healthy controls
	*E2F1*; 20q11.22	Li *et al*. [64]	Sporadic	Cross-sectional	Biological assays, including cell cycle, viability, apoptosis, immunofluorescence, RT-PCR, and western blotting	Chinese population: unilateral and bilateral 15 DDH cases and 12 healthy
	*UQCC*; 20q11.22 and rs6060373	Sun *et al*. [51]	Sporadic	Case–control	GWAS	386 DDH cases and 558 healthy
22	*PPP6R2*; 22q13.33	Dembic *et al*. [77]	Familial	Cross-sectional	WES	Danish population: 29 families examined including 66 DDH cases

a Reverse Transcriptase Polymerase Chain Reaction.

b Gene Set Enrichment Analysis.

**Table 2.** Genes implicated in FAI from literature review.

Chromosome	Gene(s); location	Study	Inheritance details	Study type	Method	Population
1	*ADAMTS4*; 1q23.3	Kuhns *et al*. [62]	Sporadic	Cross-sectional	Whole-genome RNA sequencing	10 patients with FAI (Tönnis angle > 60°) and 10 patients with osteoarthritis secondary to FAI; 17 patients for validation analysis (a total of 37 patients)
		Hashimoto *et al*. [92]	Familial and sporadic	Case–control	RT-PCR^a^	32 FAI patients, 7 osteoarthritis patients, 3 healthy controls
		Chinzei *et al*. [63]	Sporadic	Cross-sectional	RT-PCR	30 FAI patients and 30 osteoarthritis patients
4	*CXCL1, CXCL3*, and *CXCL6*; 4q13.3	Hashimoto *et al*. [92]	Familial and sporadic	Case–control	RT-PCR	32 FAI patients, 7 osteoarthritis patients, and 3 healthy controls
	*IL-8*; 4q13.3					
7	*IL-6*; 7p15.3	Gao *et al*. [70]	Sporadic	Cross-sectional	RT-PCR	Bone samples from 12 cam-type FAI patients and 6 healthy patients
8	*TNFRSF11B*; 8q24.12	Gao *et al*. [70]	Sporadic	Cross-sectional	RT-PCR	Bone samples from 12 cam-type FAI patients and 6 healthy patients
11	*MMP13*; 11q22.2	Kuhns *et al*. [62]	Sporadic	Cross-sectional	Whole-genome RNA sequencing	10 patients with FAI (Tönnis angle > 60°) and 10 patients with osteoarthritis secondary to FAI; 17 patients for validation analysis (a total of 37 patients)
		Hashimoto *et al*. [92]	Familial and sporadic	Case–control	RT-PCR	32 FAI patients, 7 osteoarthritis patients, and 3 healthy controls
		Chinzei *et al*. [63]	Sporadic	Cross-sectional	RT-PCR	30 FAI patients and 30 osteoarthritis patients
12	ALPL; 1p36.12	Gao *et al*. [70]	Sporadic	Cross-sectional	RT-PCR	Bone samples from 12 cam-type FAI patients and 6 healthy patients
	COL2A1; 12q13.11	Hashimoto *et al*. [92]	Familial and sporadic	Case–control	RT-PCR	32 FAI patients, 7 osteoarthritis patients, and 3 healthy controls
13	TNFSF11; 13q.14.11	Gao *et al*. [70]	Sporadic	Cross-sectional	RT-PCR	Bone samples from 12 cam-type FAI patients and 6 healthy patients
15	*ACAN*; 15q26.1	Hashimoto *et al*. [92]	Familial and sporadic	Case–control	RT-PCR	32 FAI patients, 7 osteoarthritis patients, and 3 healthy controls
		Chinzei *et al*. [63]	Sporadic	Cross-sectional	RT-PCR	30 FAI patients and 30 osteoarthritis patients
17	*HOXB9*; 17q21.32, rs8844, rs3826541, rs3826540, rs7405887, and rs79931349	Sekimoto *et al*. [93]	Sporadic	Case–control	GWAS	Japanese population: 130 FAI patients and 186 healthy controls
	*CCL3L1*; 17q21.1	Hashimoto *et al*. [92]	Familial and sporadic	Case–control	RT-PCR	32 FAI patients, 7 osteoarthritis patients, and 3 healthy controls
	*CCL3*; 17q12	Hashimoto *et al*. [92]	Familial and sporadic	Case–control	RT-PCR	32 FAI patients, 7 osteoarthritis patients, and 3 healthy controls

a Reverse Transcriptase Polymerase Chain Reaction.

### Genes related to DDH and osteoarthritis

#### FRZB (2q32.1)

*FRZB* encodes the frizzled-related protein that serves as an antagonist in the WNT signalling pathway. This protein has a role in regulating skeletal morphogenesis, chondrocyte differentiation, and cartilage homeostasis and is associated with occult DDH and osteoarthritis development, particularly in female probands [30, 31].

#### CX3CR1 (3p22.2)

*CX3CR1* encodes C-X3-C motif chemokine receptor 1, a G-protein-coupled receptor for CX3CL1. This gene has a role in cell adhesion, differentiation, and proliferation of mesenchymal cells or osteoblasts. There are several alleles that have been associated with DDH, including rs3732378 and rs3732379. Additionally, CX3CR1 expression has been identified in synovial membrane specimens of patients with both osteoarthritis and rheumatoid arthritis [32, 33]. Gumus *et al*. revealed a 12-fold and 75-fold increased risk of DDH in recessive and dominant modelling of the CX3CR1 rs3732378 polymorphism in a Turkish population [34]. Feldman *et al*. examined a four-generational family with DDH and found a variant of *CX3CR1* shared by all affected members [35]. Basit *et al*. examined the missense variant rs3732379; however, they found it did not segregate with DDH in a family and did not have a functional effect on the encoded protein. Additionally, this variant is frequent in the general population, suggesting low penetrance, although it may be a modifier of the DDH phenotype [36].

#### ASPN (9q22.31)

*ASPN* encodes an extracellular matrix (ECM) protein. *ASPN* inhibits the formation of fascia, tendons, and cartilage via the Transforming Growth Factor Beta (TGF-β) and BMP2 pathways. Shi *et al*. conducted a case–control study on Chinese individuals, which associated a 14 D-repeat (D14) polymorphism in this gene with DDH [37]. Kizawa *et al*. also showed that overexpression of the D14 allele has also been observed in patients with hip and knee osteoarthritis [38]. Interestingly, this D-repeat is implicated in knee osteoarthritis in Asians, but not in Europeans [37].

#### DKK1 (10q21.1)

*DKK1* encodes a secreted protein with two cysteine-rich domains that mediate protein interactions. The DKK1 protein binds to the LRP6 co-receptor to inhibit the beta-catenin-dependent Wnt signalling, playing a role in chondrogenesis, osteogenesis, joint development, and articular cartilage homeostasis. Liu *et al*. found that a variant rs1569198 of *DKK1* is associated with a three-fold increase in developing DDH in a Chinese Han female population [39]. This gene has also been implicated in osteoarthritis and inflammatory arthritis via its correlation with inflammatory cytokine levels and pro-apoptotic regulators [40].

#### PDRG1 (20q11.21)

*PDRG1* encodes a protein that acts as a subunit in multiple macromolecular complexes. It is found in RNA polymerase II, and splicing and nutrient sensing machinery. *PDRG1* serves as a substrate for methionine adenosyltransferase, resulting in epigenetic methylation of DNA and histones. A study of 770 cases of patients with DDH from the UK found a significant positive correlation of *PDRG1* with DDH susceptibility using a linkage disequilibrium score to determine *PDRG1* overlap between DDH and osteoarthritis (*P* = .0047) [41].

#### GDF5 (20q11.22)

*GDF5* encodes a ligand for TGF-β. This protein regulates osteogenesis, joint development, and endochondral ossification [42]. Patients with osteoarthritis have upregulated *GDF5* in cartilage relative to healthy controls [43]. Osteoarthritis has been associated with the presence of the T allele in rs143383, located in the promoter region of *GDF5*, resulting in reduced transcription [44]. This gene has been identified as a risk factor for DDH in French, Chinese, Saudi Arabian, and Greek populations [41, 45–47]. SNPs rs143383 and rs143384 have been specifically implicated in the pathogenesis of DDH, knee osteoarthritis, and lumbar-disc degeneration [48]. An analysis of 45 patients with DDH and 45 matched controls identified a significantly higher rate of hypermethylation of the *GDF5* promoter region in the diseased cohort [49]. Novakov *et al*. identified a correlation between rs143384 and osteoarthritis in nonobese patients [50]. Sadat-Ali *et al*. found a correlation between rs143383 and DDH in children [45].

#### UQCC1 (20q11.22)

*UQCC1* encodes a zinc-binding protein involved in morphogenesis and growth of the skeleton. Sun *et al*. identified 12 variants in *UQCC* associated with DDH in a Han Chinese population [51]. They replicated their findings by looking specifically at rs6060373, one of the 12 variants, in two distinct populations with both case and control groups conformed to the Hardy–Weinberg equilibrium. Results from the first and second populations supported their hypothesis that *UQCC* is a risk locus for DDH.

#### TGF-β1 (19q13.2)

*TGF-β1* encodes a secreted ligand of the TGF-β superfamily of proteins. TGF-β plays a crucial role in chondrogenesis via activation of Smad, ALK, and TGF family receptors. A case–control study noted that cases of osteoarthritis secondary to DDH were more likely to have homozygosity of *TGF-β1* locus29 rs1800470 than those with osteoarthritis unrelated to DDH within Caucasians of European descent [52]. Fava *et al*. identified elevated levels of active TGF-β in osteoarthritic patients compared to their healthy counterparts [53]. Mechanical joint loading causes transient activation of TGF-β in articular cartilage. However, in contrast to Fava *et al*.’s findings, Vanwanseele *et al*. showed that mechanical joint loading benefits articular cartilage. Based on this, one can infer that transient joint loading, and the temporary activation of TGF-β, may help protect against osteoarthritis [54]. Smad3, a TGF-β receptor, when defective, causes early-onset osteoarthritis in humans [55]. High levels of TGF-β, as seen in osteoarthritis, cause preferential activation of the Smad1/5/8 pathway, which results in chondrocyte activation and hypertrophy [56–59]. Ma *et al*. identified SNP rs1800470 in a population of Han patients and found that it was significantly related to the risk of DDH [60].

### Gene related to FAI and osteoarthritis

#### ADAMTS4 (1q23.3)

*ADAMTS4* encodes an enzyme that degrades aggrecan within the ECM of cartilage, which is particularly important in arthritic disease progression [61]. Kuhns *et al*. examined upregulated expression of *ADAMTS4* in the cartilage of patients with high-grade FAI and osteoarthritis [62]. Chinzei *et al*. found higher mRNA expression of *ADAMTS4* in the cartilage of 30 patients with FAI undergoing hip arthroscopy compared to 30 patients with osteoarthritis undergoing THA. They also found greater gene expression of *ADAMTS4* in FAI patients with larger alpha angles, particularly those ≥60° [63].

### Genes related to DDH, FAI, and osteoarthritis

#### COL1A1 (17q21.33)

*COL1A1* encodes the proalpha-1 chains of type I collagen, found in bone, connective tissues, and tendon. This gene plays an important role in chondrogenesis, osteogenesis, and ligament formation. Li *et al*. identified >1000 genes that were differentially expressed in the hip joint capsules of 15 patients with DDH versus 12 healthy controls. In affected patients, *COL1A1* was downregulated [64]. A case–control study also identified three variations in the *COL1A1* promoter among 10 of 154 Chinese female patients with DDH that were not found in any of the 180 matched healthy controls [65]. In another study that used snRNA and MiP sequencing to analyse ligamentum teres samples in six patients undergoing open reduction for DDH, *COL1A1* was shown to have a role in tissue thickening and hypertrophy [66].

Studies have shown that the expression of *COL1A1* increases significantly with higher grades of osteoarthritis severity in cartilage tissue [67]. Notably, the ratio of *COL2A1* (which encodes type II collagen) to *COL1A1* expression drops dramatically as osteoarthritis progresses, indicating a shift towards a more fibroblastic phenotype in chondrocytes. *COL1A1* has been identified as one of the upregulated differentially expressed genes in osteoarthritic cartilage, suggesting its role in mediating sensitivity to disease progression [68]. The increased expression of *COL1A1*, along with *COL2A1*, is thought to reflect the metabolic activation of chondrocytes in response to the osteoarthritis process. Chinzei *et al*. found increased expression of *COL1A1* in the labrums of 30 patients with osteoarthritis compared with 30 patients with FAI [63].

#### MMP13 (11q22.2)

*MMP13* encodes proteins involved in the breakdown of type II collagen in the ECM, as well as cartilage turnover. Breakdown of ECM is thought to play an important role in the pathogenesis of osteoarthritis. Davidson *et al*. compared hyaline surface protein content in patients receiving THA for osteoarthritis versus hip fractures and found that the MMP family was significantly upregulated in arthritic patients [69]. Li *et al*. found that the expression of *MMP13* was downregulated in the hip joint capsules of Chinese patients with DDH compared to healthy controls [64]. Kuhns *et al*. compared matrix metalloproteinase activity in osteoarthritis versus FAI and found elevated levels of MMP13 protein in cartilage of patients with late-stage FAI and osteoarthritis [62]. Chinzei *et al*. found higher mRNA expression of *MMP13* in the cartilage of 30 patients with FAI undergoing hip arthroscopy compared to 30 patients with osteoarthritis undergoing THA [63].

#### IL-6 (7p15.3)

The *IL-6* gene encodes pro-inflammatory cytokine IL-6, which promotes the production of matrix metalloproteinases and other catabolic factors that contribute to cartilage degradation. Gao *et al*. found increased expression of *IL-6* in bone tissue samples of 12 patients with early-stage cam-type FAI compared to those of healthy controls [70]. A case–control study found rs1800796 was associated with an increased risk of developing osteoarthritis secondary to DDH compared to patients with osteoarthritis unrelated to DDH in a population of Caucasians of European descent [52]. Numerous studies have further reported elevated levels of IL-6 in the synovial fluid, cartilage, and serum of osteoarthritis patients. Additionally, IL-6 levels positively correlate with radiographic severity and progression of osteoarthritis [71–75].

### Other genes related to DDH/FAI, but with a limited association with human osteoarthritis in the literature

Several other genes are implicated in DDH but currently have a sparse association with human osteoarthritis in the literature, including BMS1, HOXD9, TBX4, TENM3, and PAPPA2. HOXB9 is implicated in both FAI and DDH but also lacks a strong association with human osteoarthritis.

## Discussion

Degenerative hip disease affects >27 million Americans, causing pain and reduced quality of life at an annual cost of tens of billions of dollars [94, 95]. Pathologies including DDH and FAI share a critical pathway of altering hip morphology, resulting in abnormal biomechanics for the remainder of a patient’s life and increasing their risk for early-onset osteoarthritis. This scoping review was conducted to elucidate the genetic factors underlying MHA and its association with osteoarthritis.

A broad spectrum of populations was examined. Eleven studies examined Asian cohorts, seven Middle Eastern, six European, four North American, and only one African cohort (South Africa), which was predominantly of European descent. Twelve articles examined cohorts with sporadic inheritance, six with familial inheritance, and eight with sporadic and familial cases. Only two studies focused exclusively on unilateral DDH; the remaining studies assessed unilateral and bilateral cases.

Twenty-one studies, in addition to several review and narrative articles, reported on the genetic profile of DDH. The studies employed GWAS, gene set enrichment analyses (GSEA), candidate gene association studies, WES, transcriptome analysis, biological assays, Sanger sequencing, Reverse Transcriptase Polymerase Chain Reaction (RT-PCR), and bioinformatics. Several genes have been implicated, with frequent associations found for *GDF5, COL2A1, COL1A1, HOXB9*, and *CX3CR1*. Genes involved in ECM components, bone/cartilage development, and signalling pathways like TGF-β and Wnt have been commonly associated with DDH and osteoarthritis. The studies were conducted on diverse populations, including Chinese, Japanese, Caucasian, Turkish, Saudi Arabian, and Danish groups, with sample sizes ranging from small families to GWAS cohorts with thousands of participants.

Only five studies in the review discussed the genetic profile of FAI. The studies employed research methods including whole genome RNA sequencing, GWAS, and RT-PCR. Commonly implicated genes include *ADAMTS4* and *ACAN. ADAMTS4* is also involved in the pathogenesis of osteoarthritis. This gene encodes an enzyme that degrades aggrecan and collagen in the ECM of articular cartilage. *ACAN* provides instructions for making aggrecan.

Genes related to FAI, DDH, and osteoarthritis include *COL1A1, MMP13*, and *IL-6. MMP13*, like *ADAMTS4*, encodes an enzyme that degrades aggrecan and collagen within articular cartilage. *COL1A1*, involved in chondrogenesis, osteogenesis, and ligament formation, has increased expression in these conditions, likely reflecting the metabolic activation of chondrocytes in response to cartilage breakdown. Finally, *IL-6* encodes pro-inflammatory cytokine IL-6, which promotes the production of matrix metalloproteinases and other catabolic factors that contribute to cartilage degradation.

This complex interplay between signalling pathways, transcriptional regulators, ECM components, and inflammatory processes highlights the multifactorial nature of MHAs, involving various biological mechanisms that contribute to the abnormal hip joint formation and the pathogenesis of osteoarthritis. Several other genes implicated in DDH and/or FAI but with a sparse association with human osteoarthritis in the literature, including *BMS1, HOXD9, TBX4, TENM3, PAPPA2*, and *HOXB9*, should be further investigated to elucidate their role in osteoarthritis development.

Morphologic abnormalities are typically present early in life, well before symptoms develop. Painful symptoms result only after years or decades of damage potentiated by kinematic conflict that could have been altered before irreversible cartilage damage occurs [96]. Thus, critical windows exist for the identification of pathologic morphologies and early intervention as indicated. This proof-of-concept has been well demonstrated in countries such as Germany and Austria, which have instituted universal ultrasound screening for infant DDH and essentially eradicated late-presenting DDH sequelae [97]. Patients identified to have DDH or FAI are treated in an age-appropriate way with bracing or surgical intervention (arthroscopy, osteotomies, etc.) to reduce the rates of adult hip osteoarthritis [98]. These age- and disease-specific treatment strategies have been developed for these conditions to alter the natural history of hip degeneration with the hopes of preventing osteoarthritis. Unfortunately, unlike many other disease processes, modern medicine has yet to identify a method of disease reversal for hip osteoarthritis; once it progresses to a moderate or severe stage, THA is the only definitive treatment option. As such, morphological screening based on multimodal assessments could provide new guidance for the early treatment of osteoarthritis, subsequently reducing the overall healthcare burden of hip osteoarthritis.

MHAs span a diverse spectrum of pathologies, extending far beyond the scope discussed in the current review, which predominantly focuses on FAI and DDH. A simplified paradigm of MHAs as asphericity and/or under- or over-coverage of the femoral head guided the present review. However, as research advances, a more nuanced understanding of 3D morphology and its relationship with genetics and osteoarthritis is anticipated. Contemporary methods for image analysis, such as SSM, have emerged as powerful tools to better capture complex 3D anatomical structures [29, 99–102]. Future studies should explore their correlation with genetic fingerprints as was performed in a number of articles that populated in the current review [27–29]. Other morphological conditions, such as SCFE and Legg–Calvé–Perthes, should be explored for a more comprehensive understanding of MHAs and implications for the progression to osteoarthritis.

The integration of genetic studies, advanced imaging techniques, and an understanding of biomechanical and inflammatory pathways offers a comprehensive approach to managing MHAs. By identifying at-risk individuals through genetic and clinical assessments, personalized treatment strategies can be developed to prevent or delay the onset of symptomatic osteoarthritis. As research continues to evolve, particularly in the realm of genetic markers and their role in hip morphology and osteoarthritis, there is great potential to improve patient outcomes and enhance the quality of life for those affected by degenerative hip disease. The incorporation of these multimodal assessment tools into clinical practice could pave the way for more effective, early interventions, ultimately transforming the landscape of hip disease management.

## Supplementary Material

hnaf020_Supp

## Data Availability

The data was derived from sources in the public domain.

## Appendix

The following query was entered into PubMed search criteria: ‘((“genomics”[Title/Abstract] OR “genetics”[Title/Abstract] OR “genes”[Title/Abstract]) AND (“femoroacetabular impingement”[Title/Abstract] OR “developmental dysplasia of the hip”[Title/Abstract] OR “dysplasia”[Title/Abstract] OR “hip morphology”[Title/Abstract])) AND ((“genomics”[Title/Abstract] OR “genetics”[Title/Abstract] OR “genes”[Title/Abstract]) AND (“FAI”[Title/Abstract] OR “developmental dysplasia of the hip”[Title/Abstract] OR “hip dysplasia”[Title/Abstract] OR “impingement”[Title/Abstract] OR “hip morphology”[Title/Abstract]))’.
