# Supplementary material for: Genetics of morphological hip abnormalities and their implications for osteoarthritis: a scoping review
Source: J Hip Preserv Surg. 2025 Apr 18;12(3):202–16. doi: 10.1093/jhps/hnaf020 (PMC12461200; doi:10.1093/jhps/hnaf020)
Supplement: hnaf020_Supp [file hnaf020_supp.zip › suppl_data/Appendix.docx]

Appendix:

The following query was entered into Pubmed search criteria:

“(("genomics"[Title/Abstract] OR "genetics"[Title/Abstract] OR "genes"[Title/Abstract]) AND ("femoroacetabular impingement"[Title/Abstract] OR "developmental dysplasia of the hip"[Title/Abstract] OR "dysplasia"[Title/Abstract] OR "hip morphology"[Title/Abstract])) AND (("genomics"[Title/Abstract] OR "genetics"[Title/Abstract] OR "genes"[Title/Abstract]) AND ("FAI"[Title/Abstract] OR "developmental dysplasia of the hip"[Title/Abstract] OR "hip dysplasia"[Title/Abstract] OR "impingement"[Title/Abstract] OR "hip morphology"[Title/Abstract]))”
